# Psychological, Physical, and Heat Stress Indicators Prior to and after a 15-Minute Structural Firefighting Task

**DOI:** 10.3390/biology11010104

**Published:** 2022-01-10

**Authors:** Elisa F. D. Canetti, Scott Gayton, Ben Schram, Rodney Pope, Robin M. Orr

**Affiliations:** 1 Tactical Research Unit, Bond University, Gold Coast 4226, Australia; gayton.scott2@gmail.com (S.G.); bschram@bond.edu.au (B.S.); rpope@csu.edu.au (R.P.); rorr@bond.edu.au (R.M.O.); 2 Faculty of Health Sciences and Medicine, Bond University, Gold Coast 4226, Australia; 3 School of Allied Health, Exercise and Sports Sciences, Charles Sturt University, Albury-Wodonga 2640, Australia

**Keywords:** firefighters, cognition, fatigue, motivation

## Abstract

**Simple Summary:**

Firefighters must endure extreme environments. Such exposure increases their body temperature, which can induce fatigue, reduce motivation, and impair their decision-making. This study set out to investigate the relationship between these factors. Nine firefighters were required to complete simulated firefighting tasks in a controlled structural fire for 15 min. Logical reasoning, speed and accuracy, memory recall, general motivation and fatigue, and physical and mental effort were recorded prior to, immediately after, and 20 min after the simulation. Results of this study identified that alongside a significant increase in firefighter tympanic membrane temperature post-task; (1) body weight loss was poorly correlated with post-task motivation and fatigue scores; (2) pre-task logical reasoning scores were predictive of change in tympanic membrane temperature.

**Abstract:**

Firefighters work in strenuous conditions for prolonged periods wearing up to 20 kg of personal protective equipment. This often contributes to significant heat and cardiovascular strain. This study examined the relationships between psychological and physical measures taken prior to undertaking a 15 min firefighting task, and the occurrence of heat stress and high levels of fatigue following the task. Nine qualified firefighters completed a 15 min “live burn” scenario designed to mimic a fire started by a two-seater couch in a lounge room and completed simulated tasks throughout the duration. Logical reasoning, speed and accuracy, general motivation and fatigue, and physical and mental effort were recorded pre-scenario, and at 0- and 20-min post-scenario. General motivation and fatigue scores at 0- and 20-min post-scenario were highly correlated with each other (r_s_ = 0.90; *p* = 0.001). The general motivation and fatigue scores, at 0- and 20-min post-scenario, were also strongly related to pre-task logic/reasoning test scores (Post 0 r_s_ = −0.77, *p* = 0.016; Post 20 r_s_ = −0.87, *p* = 0.002). Firefighters with lower logical reasoning and speed and accuracy scores were more susceptible to fatigue and impaired cognition when exposed to rises in core temperature and heat stress.

## 1. Introduction

Firefighters work for prolonged periods in strenuous conditions, often in high ambient temperatures [1]. In operational environments, firefighters perform strenuous tasks including victim search and rescue, stair and ladder climbing, and carriage of heavy equipment. These commonly performed tasks have a high-energy-expenditure cost [1,2]. The energic cost of these tasks can be further burdened by the extreme temperatures and the additional weight (approximately 20 kg) of the Personal Protective Equipment (PPE) worn [3,4,5]. It has been well documented that the strenuous work firefighters engage in under these conditions contributes to significant cardiovascular and thermal strain [2,5,6,7]. 

Thermal strain is commonly observed in firefighters. The use of PPE negatively impacts thermoregulation by preventing heat dissipation [6], and reduced fluid intake during fire suppression promotes dehydration [1,8,9,10]. Additionally, re-entry to the fire after short periods of passive recovery extends exposure periods, compounding thermal strain [11]. Increases in exposure periods to heat have been shown to further decrease perceptual capabilities, motor responses, and cognitive attributes [2,12]. Additionally, heat exposure and dehydration increase subjective perception of the difficulty of exertion, which interferes with attention, vigilance, short-term memory, working memory, stress response, and psychomotor skills [10,13,14]. Despite such findings, the impact of thermal strain and the inherited stress of fire suppression on physical and cognitive function of firefighters following heat exposure is still to be fully elucidated. 

Simulation of a wildland fire at 45 °C demonstrated that, despite the increase in firefighter’s core temperature, no cognitive decline was observed [15]. The authors postulated that maintenance of the firefighters’ hydration status may have preserved their cognitive function. However, accessibility to, or opportunity for, fluid intake during fire suppression is not always guaranteed. Conditions vary significantly based on the location of the fire, with recent research indicating that firefighters consider structural firefighting as the hottest operational environment [16]. Exposure to structural fires of 30 min to 1 h of duration results in some level of dehydration, regardless of fluid intake allowance [1,9,10,17]. Still, little is known about how the physiological adaptations to the intense heat exposure during structural fires impact on decision-making and cognition function of firefighters. Therefore, the current pilot study aims to examine the relationships between psychological and physical measures taken prior to a 15 min structural firefighting task and the occurrence of heat stress and high levels of fatigue following the task. 

## 2. Materials and Methods

The current study was conducted concurrent to, and using, the same participants and tasks as a previously published study of dehydration in firefighters [18]. However, additional measures were taken to achieve this study’s aim.

### 2.1. Participants 

A sample of nine firefighters (seven males and two females) were recruited from the Queensland State Fire and Emergency Service to participate in a “live” structural firefighting scenario. Firefighters were included if they (1) were fully qualified, (2) were fit for normal duty, (3) were cleared for participation by their supervisor, and (4) consented to participation. There were no exclusion criteria. 

Participant characteristics are listed in Table 1. Standard authorised PPE, Australian Defence Apparel Firefighter Garments Structural Uniforms (mean weight 21.4 ± 0.7 kg) and a Scott Advanced Carrying System Singe Cylinder (6.8 L), Self-Contained Breathing Apparatus (SCBA) (weighing 9.6 kg full; 8.6 kg empty) were worn by all personnel for the duration of the scenario.

### 2.2. Data Collection 

Data were collected at a Fire and Emergency Service live fire training facility in Queensland, Australia. 

### 2.3. Scenario

Firefighters were separated into two teams of four and five participants for convenience in managing the scenarios, but data for the two groups were pooled for analyses. Each firefighter undertook pre-scenario body weight and tympanic membrane temperature testing, prior to completing a set firefighter task in the scenario (outlined below). Prior to these tests, they also undertook a 60 s speed and accuracy test, a 30 s logical reasoning test, and a short memory test, all of which are further described below. 

Two tasks were set up, one after the other, on-site and in a single burn chamber. The on-site scenario comprised a burn “load” within a designated structure, equivalent to a standard two-seater couch fire. Inside the structural fire, two tasks simulating typical firefighting duties were arranged. Both tasks were 7.5 min in duration and consisted of (1) a low-posture victim drag with an 80 kg dummy along a designated five-meter area; (2) a hose drag along a designated five-meter area. Once firefighters had completed the task, they moved down and back into a crouched position at a slow, deliberate pace. 

The scenarios lasted for a total of 15 min (limited by oxygen cylinder capacity), with firefighters swapping tasks on the command of the Safety Officer with the “burn” chamber after 7.5 min. Firefighters exited the “burn” chamber after completion of the final task, proceeding directly to tympanic membrane temperature measurement, followed by assessments of perceived physical and mental effort during the task, general motivation and fatigue, speed and accuracy, logical reasoning, and memory recall; all further described below. At 20 min post-scenario, each firefighter undertook the same testing battery again, completed in the above order, except for the perceived effort scales. Team one completed the scenario first, with team two completing the scenario 30 min later after the structure was refuelled. This ensured that the structure reached the same thermal profile that team one was exposed to. Team two completed pre- and post-simulation measures as team one did. 

The average fire temperature was 40.0 °C (maximum 50.9 °C) at 0.3 m above the floor, and 458.3 °C (maximum 571.5 °C) at the ceiling level (approx. 2.6 m). The fire was maintained at 130–155 °C at 1.1 m above the floor. The mean relative humidity within the burn chamber during the scenario was 53% (maximum 59%). The ambient environmental temperature was 24.8 °C, with a relative humidity of 87%.

Two firefighting Safety Officers were positioned appropriately throughout the scenario to guide tasks and ensure safety of all personnel. Ethical approval for this research was granted by Bond University Human Research Ethics Committee (Protocol number RO1761). 

### 2.4. Testing Protocol

Outcome measure data were collected pre-scenario, immediately post-scenario (0 min), and at 20 min post-scenario. The researchers involved in data collection conducted the outcome measures using the same sequence at each timepoint, and to avoid any potential inter-rater bias, the same researcher was responsible for a given outcome measure, throughout. 

Following pre-scenario testing, fluid or food consumption was prohibited (unless directed for medical reasons) until final testing was completed. Throughout the recovery period, firefighters remained in their PPE. This replicated real-life scenarios where firefighters exit a structural fire for short periods before possibly re-entering and ensured standardization of the protocol. A description of the testing protocol is outlined below.

#### 2.4.1. Tympanic Membrane Temperature

Tympanic membrane temperature measurements have previously been used in the field after recovery from intense fire operations [19] as a relatively non-invasive approximation of core temperature. Tympanic temperature was measured under the flash hood (to reduce impacts of ambient conditions on tympanic temperatures) of the left auditory canal using a Liberty ET-100A thermometer (Liberty Health Products, Mount Waverley, VIC, Australia). This was recorded in degrees Celsius to the nearest 0.1 degree.

#### 2.4.2. Physical and Mental Effort Scale

Two questionnaires were utilised to measure physical and mental effort, respectively. Both questionnaires, along with the general motivation and fatigue scale, were intended to determine the level of motivation/fatigue following the task.

1. Physical effort (rate of perceived exertion, RPE [20]): firefighters were asked to rate their level of physical exertion on a scale of 6 to 20, where 6 means “no exertion at all” and 20 means “maximal exertion”. The 6–20 RPE scale has a linear relationship with heart rate, providing an estimate of physical effort and exertion during physical work [21].
2. Mental effort: using the Task Effort and Awareness (TEA) score [22], firefighters were requested to think back to when they were fighting the fire and asked to rate the psychological and mental effort required to fight the fire. The TEA scale ranges from −4 to 10, where −4 mean unawareness of any mental effort and 10 constant awareness of a severe effort required to continue at the current pace and need to slow down. Different to the RPE, the TEA provides information on the psychological effort needed to continue to produce the required workload [22].

#### 2.4.3. General Motivation and Fatigue Scale 

General motivation and fatigue were measured by the Swedish Occupational Fatigue Inventory-20 (SOFI) [23] (Supplementary Figure S1) to determine how difficult firefighters found the task and the level of motivation and fatigue following the task. The SOFI has been shown to accurately portray fatigue by assessing five main dimensions: lack of energy, physical exertion, physical discomfort, lack of motivation, and sleepiness [23]. Each dimension has four expressions, totalling 20 expressions where firefighters answered spontaneously, circling a number between 0 (not at all) and 6 (to a very high degree) that best corresponded to how they felt. The firefighters reflected on the following questions: (1) How do you feel after fighting the fire? (2) To what extent do the expressions below describe how you feel? 

#### 2.4.4. Speed and Accuracy Test

The 2 and 7 test [24] (Supplementary Figure S2) was used to measure speed and accuracy. Participants were given a sheet of paper with two text blocks: (1) a series of letters with the numbers 2 and 7 scattered throughout; (2) a series of numbers with the numbers 2 and 7 scattered throughout. Participants had 90 s to cross through targets 2 and 7 as quickly as possible in both blocks. Participants were scored out of 67 possible correct targets. 

#### 2.4.5. Logical Reasoning Test

Logical reasoning (Supplementary Figure S3) was assessed using a questionnaire/quiz to examine different aspects of cognition as described by Baddeley [25]. Each participant read and answered 20 questions, systematically deciding if the description of a letter pair was either “true” or “false”. Participants had 30 s to complete as many statements as possible. 

#### 2.4.6. Memory Recall

Memory recall was assessed by using Kim’s Game, as described by Baden-Powell [26]. Participants were shown a page containing patterns of numbers, objects, pictures, and shapes (Supplementary Figure S4) for 30 s. Participants were instructed to turn the page over and required to recall what items were present. The total number of items recalled was recorded. 

Acute changes in body weight have previously been reported to reflect variation in body water content, offering a convenient measure of hydration status [27,28]. Body weight measures were conducted to assess level of dehydration and fluid loss, respectively. Body weight measures were recorded using electronic scales (BF-682W Tanita, Illinois, USA). The participants were weighed in unloaded station wear, shirts, and long pants. They were also weighed loaded whilst donning PPE pre-scenario. Unloaded body weight was reweighed at the completion of the scenario at 0 and 20 min, with loaded body weight being reweighed at 0 min. These measurements were documented to the closest 100 g. 

Environmental relative humidity and temperature were recorded from Bureau of Meteorology Brisbane Airport station measures (Bureau of Meteorology, 2016). Humidity and temperatures within the designated “burn” chamber were recorded via HMP77 Thermocouples (Vaisala Oyj, Helsinki, Finland).

### 2.5. Data Analysis 

Outcome measures were analysed descriptively, with means and standard deviations (SD) presented as appropriate. One-way repeated measures analysis of variance (ANOVA) was used to provide preliminary analysis of any changes in the measured outcomes at the three timepoints (pre-, immediately post-, and 20 min post-simulation). Due to the restricted sample size, sensitivity power analysis was performed using G*Power software (version 3.1.9.7) to determine the effect size reliably detectable. The one-way ANOVA with nine participants across three timepoints would be sensitive to effects of partial η^2^ = 0.42 with 80% power (α = 0.05). Spearman’s correlation analyses were conducted to examine the relationships between measures recorded before commencement of the fire task and post-fire task outcomes, including whether the participant withdrew from further assessment due to heat stress (this was a safety decision made by the Safety Officer on duty), change in tympanic membrane temperature, and score on the general motivation and fatigue scale. Relationships that reached statistical significance at the 0.05 or 0.10 level (two-tailed) were then graphed on scatter plots to enable visual inspection of those relationships.

## 3. Results

The simulated task challenged participants mentally and physically. There was a significant increase in tympanic temperature between the three timepoints (Table 2). The mean response score of the mental effort immediately post simulated task was 7 points (SD: 2 points), indicating a constant awareness of effort. The participants also perceived the task as hard, scoring on average 15 points (SD: 2 points) in the RPE scale. Speed and accuracy and logical reasoning scores did not change significantly between the three timepoints, but memory recall was significantly reduced post-task (Table 2). Participants lost on average 1.3% (SD: 0.2%) of their pre-fire body weight. The three participants with the highest weight loss at 20 min post-simulation (mean 1.4 kg) also had the highest increase in tympanic temperature (mean 2.6 °C) post-simulation. These participants were also the ones who reported the highest levels of fatigue at 20 min post-simulation. This indicates that for these three participants, sense of fatigue did not dissipate as quickly as it did for the others.

Two of the nine firefighter participants withdrew from further assessment in the study after completing the simulated firefighting task. One withdrew immediately after their tympanic membrane temperature was reassessed following completion of the simulated task, and one withdrew after their tympanic membrane temperature and the first series of post-task psychological measures were assessed but prior to any other physical measures being completed. In both cases, normal safety procedures were followed: the firefighter notified the Safety Officer that they were feeling heat stressed, and the Safety Officer immediately withdrew them from further participation in the study and associated tasks, directed them to usual cooling and hydration procedures, and monitored their well-being. Both participants recovered. These two participants failed to complete the general motivation and fatigue scale in three instances. In these cases, estimated scores were interpolated for each participant; participant 1 at 20 min post, and participant 2 immediately post and 20 min post. The score estimated and assigned in each of these three instances was 80 out of a possible 120 on the scale. This score was deemed a conservative estimate, since one other participant, who did not withdraw due to heat stress but was clearly fatigued, scored 84 on the scale. However, the score of 80/120 also indicated a significant level of fatigue and reduced motivation to continue participation, which would be expected of someone experiencing heat stress.

**Figure 1:**
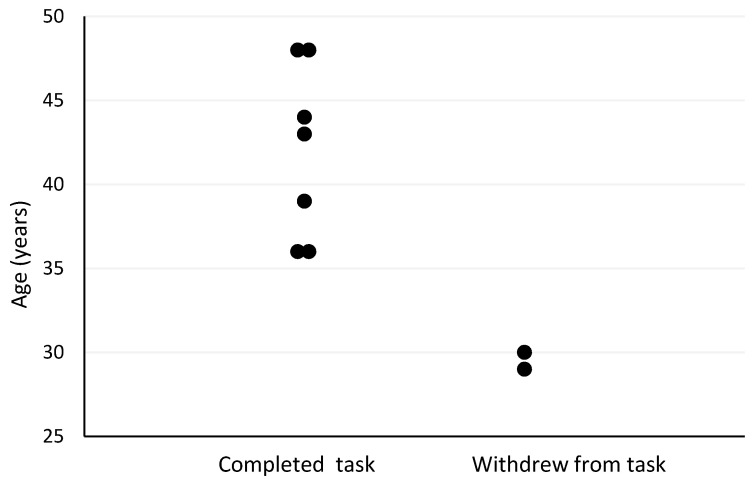
The relationship between age and withdrawal due to heat stress post-simulated firefighter activity.

**Figure 2:**
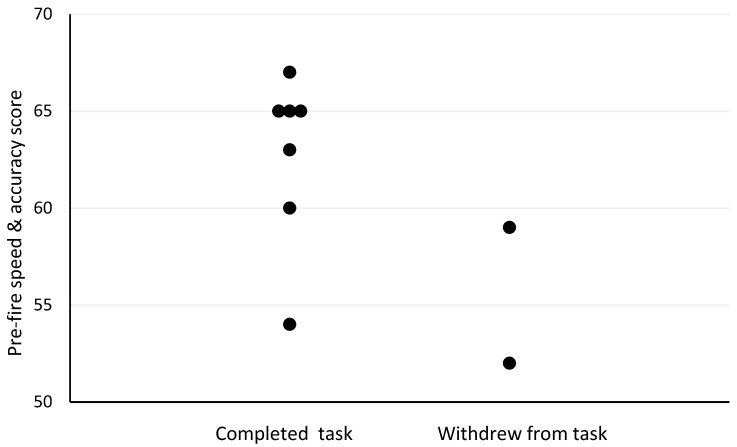
The relationship between pre-fire speed and accuracy and withdrawal due to heat stress post-simulated firefighter activity.

**Figure 3:**
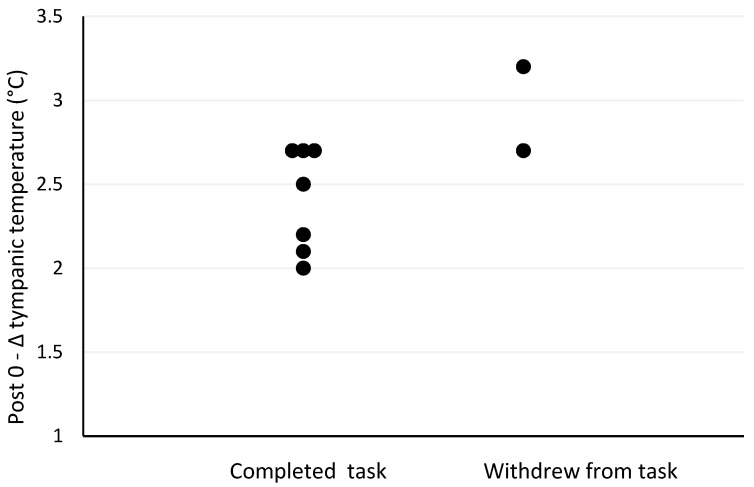
The relationship between withdrawal due to heat stress and change in tympanic temperature post-simulated firefighter activity.

**Figure 4:**
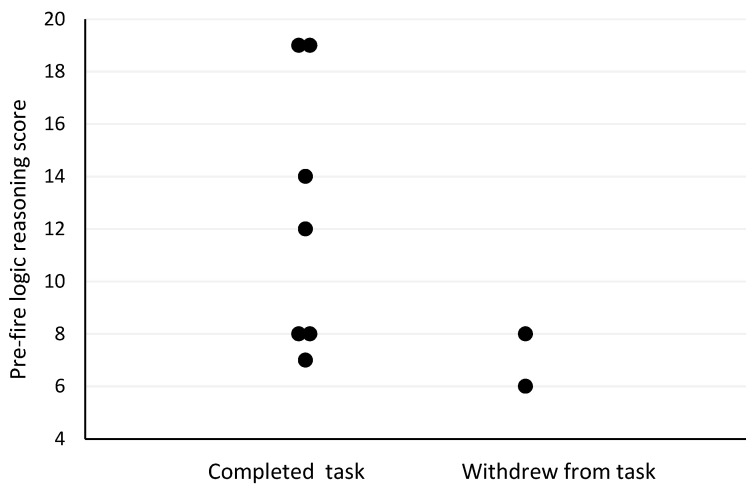
The relationship between withdrawal due to heat stress and logical reasoning pre-simulated firefighter activity.

**Figure 5:**
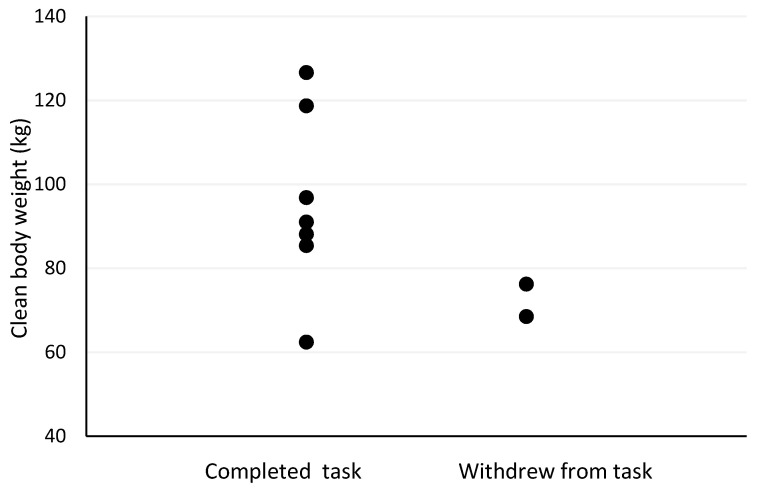
The relationship between withdrawal due to heat stress and clean body weight pre-simulated firefighter activity.

**Figure 6:**
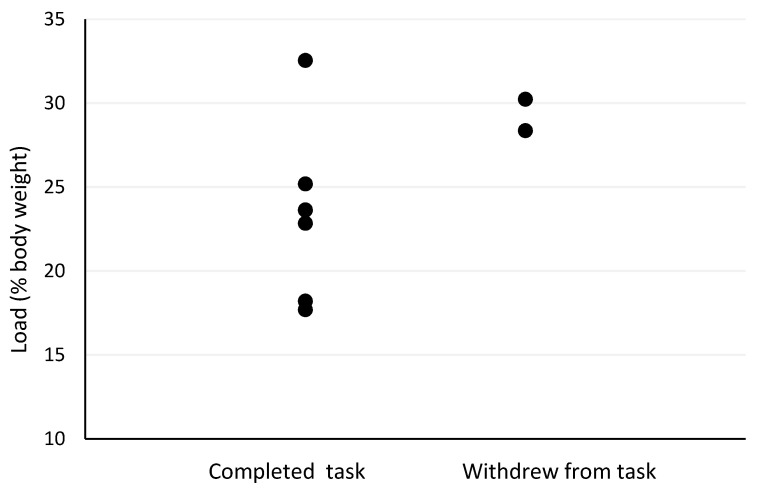
The relationship between withdrawal due to heat stress and load as a percentage of body weight pre-simulated firefighter activity.

The Spearman’s correlation analyses revealed several moderate-to-strong relationships between pre- and post-firefighting task measures and outcomes. Firefighter age was the strongest predictor of withdrawal post-firefighting task due to heat stress (r_s_ = −0.73, *p* = 0.025; Figure 1). Other moderately strong predictors of withdrawal post-firefighting task due to heat stress included pre-task scores on the speed and accuracy measure (r_s_ = −0.63, *p* = 0.068; Figure 2), change in tympanic membrane temperature post-task (r_s_ = 0.56, *p* = 0.091; Figure 3), pre-task logical reasoning scores (r_s_ = −0.53, *p* = 0.143; Figure 4), pre-task body weight (r_s_ = −0.52, *p* = 0.154; Figure 5), and PPE load as a percentage of pre-task body weight (r_s_ = −0.52, *p* = 0.154; Figure 6). 

**Figure 7:**
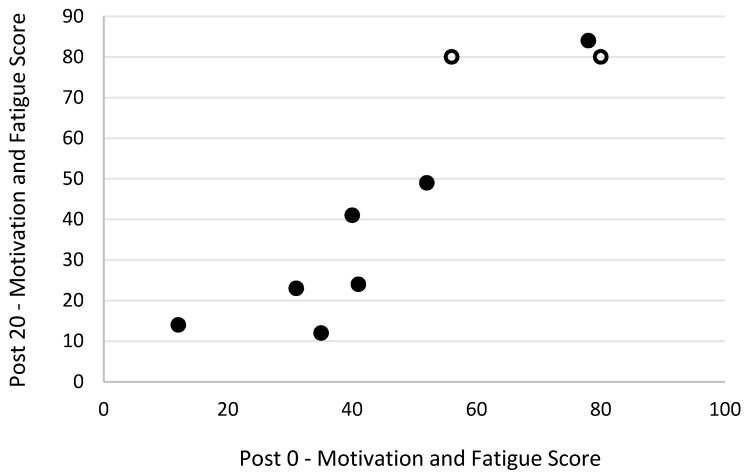
The relationship between fatigue scores immediately and 20 min post-simulated firefighter activity. Full markers (●) indicate participants who completed the task, and empty markers (O) indicate the ones that withdrew.

**Figure 8:**
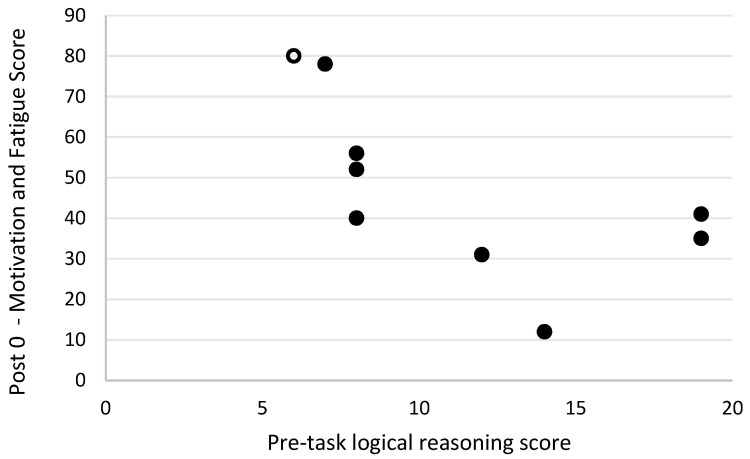
The relationship between logical reasoning score prior to simulated firefighter activity and fatigue scores immediately post-simulated firefighter activity. Full markers (●) indicate participants who completed the task, and empty markers (O) indicate the ones that withdrew.

**Figure 9:**
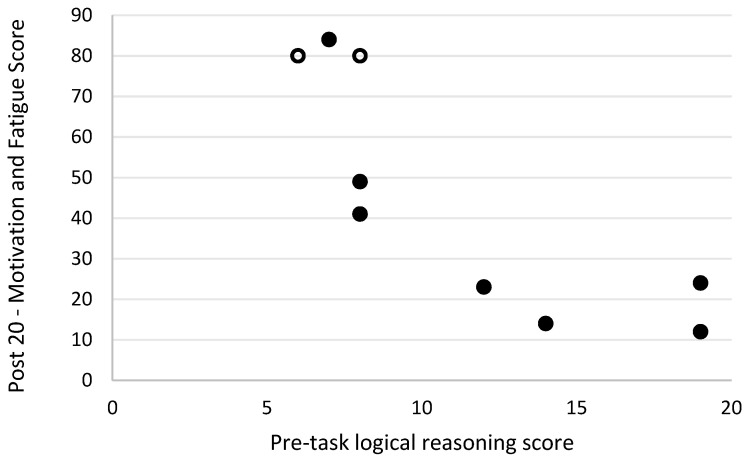
The relationship between logical reasoning score prior to simulated firefighter activity and fatigue scores 20 min post-simulated firefighter activity. Full markers (●) indicate participants who completed the task, and empty markers (O) indicate the ones that withdrew.

**Figure 10:**
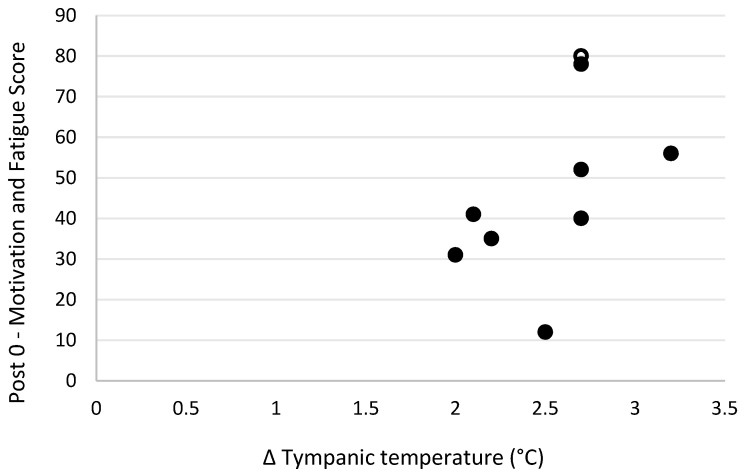
The relationship between change in tympanic temperature post-simulated firefighter activity and fatigue scores immediately post-simulated firefighter activity. Full markers (●) indicate participants who completed the task, and empty markers (O) indicate the ones that withdrew.

**Figure 11:**
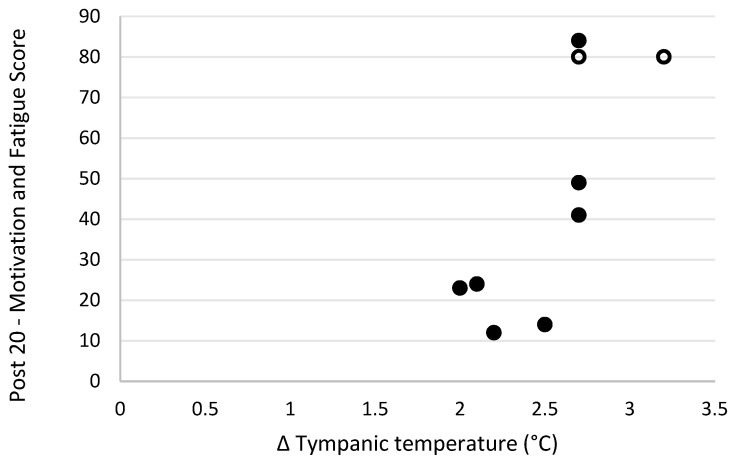
The relationship between change in tympanic temperature post-simulated firefighter activity and fatigue scores 20-min post-simulated firefighter activity. Full markers (●) indicate participants who completed the task, and empty markers (O) indicate the ones that withdrew.

**Figure 12:**
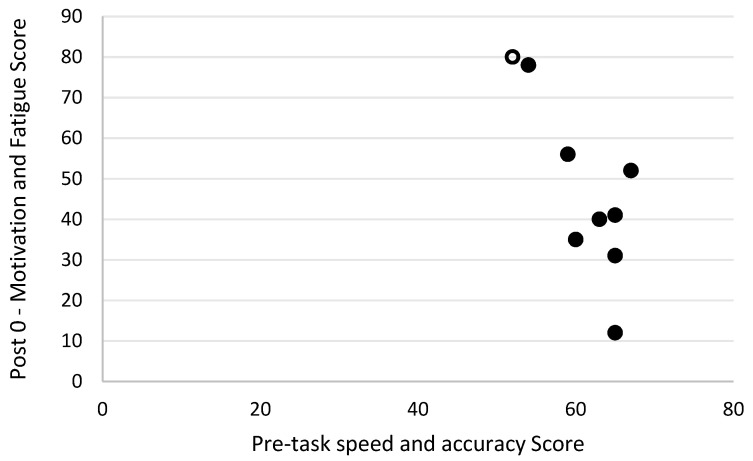
The relationship between pre-task speed and accuracy scores and fatigue scores immediately post-simulated firefighter activity. Full markers (●) indicate participants who completed the task, and empty markers (O) indicate the ones that withdrew.

**Figure 13:**
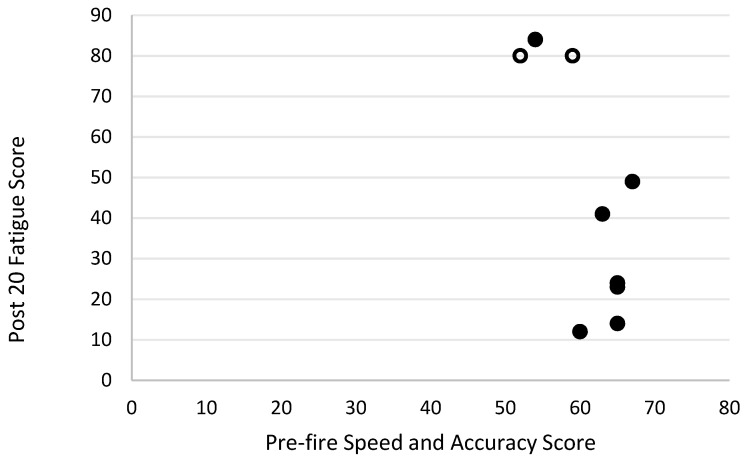
The relationship between pre-fire speed and accuracy scores and fatigue scores 20 min post-simulated firefighter activity. Full markers (●) indicate participants who completed the task, and empty markers (O) indicate the ones that withdrew.

Level of motivation and fatigue using the general motivation and fatigue scale was assessed at two timepoints following the simulated firefighting task—immediately after completion of the task and 20 min later, after a period of rest. Higher scores reflected a greater sense of fatigue and reduced motivation to continue. The general motivation and fatigue scores at these two timepoints were highly correlated with each other (r_s_ = 0.90, *p* = 0.001; Figure 7). The general motivation and fatigue scores, at both timepoints, were also strongly negatively related to pre-task logic/reasoning test scores (post 0 r_s_ = −0.77, *p* = 0.016; Figure 8, post 20 r_s_ = −0.87, *p* = 0.002; Figure 9). A strong positive association between the general motivation and fatigue scale scores and change in tympanic membrane temperature post-task was also identified (post 0 r_s_ = 0.67, *p* = 0.048; Figure 10, post 20 r_s_ = −0.76, *p* = 0.018; Figure 11). Further, the general motivation and fatigue scores were moderately negatively correlated with pre-task speed and accuracy test scores (post 0 r_s_ = −0.63, *p* = 0.071; Figure 12, post 20 r_s_ = −0.53, *p* = 0.044; Figure 13). Post-task weight loss, as an indicator of level of dehydration following the firefighting task, was poorly correlated with post-task general motivation and fatigue scores (r_s_ = −0.25; *p* = 0.585).

The strongest predictor of change in tympanic membrane temperature post-task was pre-task score on the logical reasoning test (r_s_ = −0.73; *p* = 0.026). A further moderate predictor of change in tympanic membrane temperature post-task was participant age (r_s_ = −0.56; *p* = 0.119), though again this relationship clearly did not reach statistical significance.

## 4. Discussion

The aim of this pilot study was to investigate the relationship between psychological and physical measures taken prior to undertaking a stimulated 15 min structural firefighting task and the occurrence of heat stress and high levels of fatigue following the task. The main findings of this study were, alongside significant increase in firefighter tympanic membrane temperature post-task; (1) body weight loss was poorly correlated with post-task motivation and fatigue scores; and (2) pre-task logical reasoning scores were predictive of change in tympanic membrane temperature.

Hydration is often assumed to have a significant impact on tactical personnel’s physical capacity to operate. However, preliminary results of this study suggest hydration status may be less important to operational capacity than a rise in core temperature and possibly other psychological factors are. Regarding the latter, it should be noted that this may be simply reflective of underlying physical functioning pre-task, and not a cause of differences in post-task capacity, fatigue, motivation, or heat stress.

It is known that dehydration can impair cognitive and cardiovascular function [2,29] and an individual’s ability to tolerate extreme conditions [30]. The level of dehydration induced during fire suppression is dependent on the temperature and humidity of the environment [31]. Changes in body weight post-event can be used to determine fluid loss through sweat loss. In previous studies of 30 min structural firefighting by Angerer et al. [8] and Eglin et al. [17], loss of body weight of 0.5% and 0.8% per hour, respectively, indicated that hydration was adequately maintained without fluid consumption. However, body weight loss of 1%–2% is indicative of inadequate or insufficient fluid consumption during the event [32]. Despite the weight loss reported in this study being within this range, it was poorly correlated with post-task motivation and fatigue. This suggests that hydration status did not predict or impact tolerance time or cognition in strenuous conditions. Rather, results from this study indicate fatigue is influenced more by changes in tympanic temperature than by hydration status. These results are consistent with previous research that demonstrated that the rate of heat storage was directly related to time to exhaustion [8] and that increases in heat storage reduced an individual’s ability to tolerate heat stress [33].

As predicted, firefighter’s level of fatigue immediately post-task was highly correlated with fatigue measured 20 min later. In the present study, the change in tympanic temperature predicted levels of fatigue immediately and 20 min post-task by 45% and 57%, respectively. Previous study demonstrated that firefighter’s core temperature continued to rise throughout passive recovery, after exposure to a 20 min simulated search and rescue in a heat chamber (105 °C) [11]. Walker et al. [11] highlighted that passive recovery methods were insufficient in reducing firefighter’s core temperature to the safety-recommended limit to re-enter fires. This is of note as, contrary to the present study, firefighters in the study of Walker et al. [11] were allowed to remove their PPE and consume 600 mL of ambient temperature water. Such findings indicate that even with these strategies, physical and cognitive abilities may still be impaired. Recent research has expanded on the visual and auditory cognitive impairments suffered by firefighters’ post fire suppression, highlighting significant declines in visual and auditory accuracy in cognitive tests [34]. 

Cognitive performance is influenced by motivation and fatigue [35,36]. Fatigue is known to increase reaction time, reduce alertness, and induce poor decision-making [37]; all necessary attributes to ensure safety during firefighter deployment [38]. Results from the present study indicate that a 15 min exposure to a 40−51 °C (at 0.3 m above the floor), relative humidity 53.1% environment was sufficient to induce increases in tympanic temperature and fatigue. Abbott and Schulman [39] reported that exposures to temperatures of 20−70 °C are considered routine and that firefighters are usually exposed to it for 20 min. In the present study, firefighters with the highest change in tympanic membrane temperature post-task presented with low motivation, high fatigue, and lowest speed and accuracy test scores. Reductions in speed and accuracy in cognitive test scores were also reported in occupational field research in automobile industry workers exposed to temperatures of 30−35 °C [40]. Interestingly, these researchers also reported an increase in blood concentration of adrenaline, noradrenaline, and cortisol in exposed workers [40]. In similar temperatures (36 °C), McMorris et al. [41] identified a significant correlation between changes in cortisol concentration and changes in fatigue levels from pre- to post-exposure (R^2^ = 0.48; *p* < 0.005). Reductions in higher cognitive function have been further corroborated by findings of reduced peak amplitude of P300 components of event-related potentials (ERP) measured via EEG during passive heat stress (50 °C) [42,43] and during physical activity in a hot environment (35 °C) [44]. P300 components of EPR are associated with cognitive information processing, including memory, attention, and executive function [45,46]. This is of concern as in victim rescues, for example, firefighters may be re-entering a hazardous thermal environment (70–300 °C; heat flux 1.67 and 12.56 kW/m^2^) [47] fatigued and in a state of decreased cognitive capacity, impacting their decision-making and safety.

Interestingly, scores in the pre-task logical reasoning test were predictive of changes in tympanic membrane temperature. Firefighters who scored the lowest in this test had the highest increase in tympanic temperature post-task. One possible explanation for this is the rise in stress levels in anticipation of the simulation task. Research has demonstrated that psychological stress may increase core temperature, in most cases by up to 1 °C [48,49]. However, some stressors have been shown to increase core temperature above normal body temperature (termed psychogenic fever), reaching 39 °C. Animal studies suggest that this stress-induced hyperthermia is proportional to stressor intensity [50]. Briese [49] demonstrated that preparedness and performance may be related to changes in stress-induced temperature. Low-performing students had high levels of anticipation, thus high body temperature, for both the exam (stressor) and the laboratory demonstration (control) [49]. In the present study, the two firefighters that withdrew due to increased tympanic temperature had the lowest scores in the logical reasoning test. Although these findings need to be confirmed in larger studies, it appears that, as suggested by Briese [49], management of anxiety in anticipation of the stressor may better control stress-induced hyperthermia.

The results of this study indicate some potentially valuable predictors for firefighter post-task fatigue and motivation. It has been shown in the current study that pre-scenario logical reasoning, speed and accuracy results, and age can predict those firefighters that are more likely to experience larger-magnitude rises in core temperature, heat stress, and fatigue, and reduced capacity to continue with assigned tasks. Further research with a larger sample size should aim to validate these findings and establish a model of fatigue prediction in firefighters. 

### Limitations

The current pilot study and the results are based on relatively low numbers (nine firefighters, with two withdrawing) and are thus, preliminary. Estimated scores for the general fatigue and motivation scale could have inflated the statistical power of the findings reported. As a pilot study, the inferential statistics provided here are to be interpreted with caution as the aim is solely to provide valuable information to guide future research with larger firefighter cohorts.

## 5. Conclusions

In conclusion, firefighters with lower logical reasoning and speed and accuracy scores were more likely to suffer from greater fatigue and reduced cognitive capacity due to rises in core temperature and heat stress. It is suggested that further research is required to determine the relationship of psychological, physical, and heat stress indicators with a larger cohort in simulated firefighter scenarios.

## Figures and Tables

**Table 1:** Firefighter characteristics.

Participant	Sex	Age (yrs)	Experience (yrs)	Weight (kg)
1	M	43	14	126.6
2	F	39	5	62.4
3	M	48	6	96.8
4	M	36	2	88.1
5	F	30	1	68.5
6	M	29	11	76.2
7	M	44	16	85.4
8	M	36	6	118.7
9	M	48	10	91.0
Mean	-	39.2	7.9	90.4

M = males; F = females; yrs = years; kg = kilos.

**Table 2:** Change in tympanic temperature and cognitive test scores between the three timepoints.

	Pre		0 Post		20 Post		F *	*p*	Partial η^2^
	Mean	SD	Mean	SD	Mean	SD			
Tympanic temperature (°C)	36.5	0.3	38.9	0.4	37.8	0.5	192.13	<0.001	0.97
Speed and accuracy score	62.7	4.4	64.1	2.5	65.3	0.8	2.03	0.174	0.25
Logical reasoning score	12.4	5.1	13.9	4.6	12.9	4.5	0.53	0.602	0.08
Memory recall score	6.6	1.0	8.9	1.7	3.9	1.3	19.06	<0.001	0.76

* Degrees of freedom for all RM-ANOVA = 2, 12.

## Data Availability

Data supporting the results presented here may be made available upon request.

## Supplementary Materials

The following are available online at https://www.mdpi.com/article/10.3390/biology11010104/s1, Figure S1: General Motivation and Fatigue Scale, Figure S2: Speed and accuracy test, Figure S3: Logical reasoning test, Figure S4: Memory Recall.
