# Supplementary material for: Psychological, Physical, and Heat Stress Indicators Prior to and after a 15-Minute Structural Firefighting Task
Source: Biology (Basel). 2022 Jan 10;11(1):104. doi: 10.3390/biology11010104 (PMC8773347; doi:10.3390/biology11010104)

Supplementary Figure S1 – General Motivation and Fatigue Scale

How do you feel right now?

How do you feel now after fighting the fire? To what extent do the expressions below describe how you feel?

For every expression, answer spontaneously, and circle the number that corresponds to how you feel right now.

The numbers vary between 0 (not at all) and 6 (to a very high degree).

	Not at all						To a very high degree
palpitations	0	1	2	3	4	5	6
lack of concern	0	1	2	3	4	5	6
worn out	0	1	2	3	4	5	6
tense muscles	0	1	2	3	4	5	6
falling asleep	0	1	2	3	4	5	6
numbness	0	1	2	3	4	5	6
sweaty	0	1	2	3	4	5	6
spent	0	1	2	3	4	5	6
drowsy	0	1	2	3	4	5	6
passive	0	1	2	3	4	5	6
stiff joints	0	1	2	3	4	5	6
indifferent	0	1	2	3	4	5	6
out of breath	0	1	2	3	4	5	6
yawning	0	1	2	3	4	5	6
Drained	0	1	2	3	4	5	6
Sleepy	0	1	2	3	4	5	6
overworked	0	1	2	3	4	5	6
Aching	0	1	2	3	4	5	6
breathing heavy	0	1	2	3	4	5	6
uninterested	0	1	2	3	4	5	6

Supplementary Figure S2 – Speed and accuracy test

The 2 and 7 Test

Below are two representative blocks of numbers and letters.

When told to start, you have 90 seconds to cross through Targets 2 and 7 as quickly as possible in both blocks.

Do not start until directed.

---

2 C O X C 7 1 1 J 7 H Z R I I C A S 2 Y W Q 2 L H B Z C J I I V 7 E T 2 P R V
H J H S T Q 2 C 7 K L W C 7 X H T 7 K T a 2 A V P I W O C 2 C J 7 L S 2 B I
I V W 7 T O X R 2 P H 7 P D A B I I 2 W H G A S T 2 0 P H W E D 2 T R I l
E Q x 2 P X L 7 P X 7 Z C v 7 2 Z 7 E T C H L X S D I N 7 S 2 W I S I I 7 T B I
E 0 P W H 7 P D A B I Y 2 W G F T Y 7 J

3 1 0 7 8 9 4 4 7 0 5 3 7 6 3 8 1 5 2 3 6 5 6 9 7 0 8 9 1 5 7 8 4 3 6 2 8 6
3 2 8 6 1 5 4 2 8 0 9 1 2 9 1 8 9 2 B I 3 7 6 4 5 3 7 B 0 4 6 7 9 6 2 9 1 2 8
3 9 1 8 3 7 8 9 4 6 5 9 1 4 7 0 8 6 7 1 3 0 3 9 I 0 2 3 3 8 9 4 1 2 6 5 5 3 5
7 6 B 9 5 7 0 j 9 6 1 7 3 2 8 5 9 2 8 3 1 2 B 3 3 1 4 3 B 9 4 6 2 5 3 1 0 7 8
9 4 4 7 0 5 3 7 6 3 8 1 5 2 3 6 5 6 2 8 7

Supplementary Figure S3 – Logical reasoning test

Logic Reasoning

Your task is to read each sentence and to decide whether it is a true or false description of the letter pair which follows it. If you think the sentence describes the letter pair correctly circle True. If you think the sentence does not describe the letter pair correctly, then circle False.

Start with sentence 1 and work systematically through the test without skipping any items.

You will have 90 seconds to complete as many of the statements as possible.

Work as quickly as you can without making mistakes. Do not start until directed.

1. A does not follow B	AB	True	False
2. A does not precede B	BA	True	False
3. B does not follow A	BA	True	False
4. A precedes B	AB	True	False
5. A is not preceded by B	BA	True	False
6. B is not preceded by A	BA	True	False
7. B precedes A	BA	True	False
8. B is not followed by A	BA	True	False
9. B does not follow A	AB	True	False
10. B is. not preceded by A	AB	True	False
11. B is not followed by A	AB	True	False
12. A precedes B	BA	True	False
13. A is not followed by B	AB	True	False
14. A follows B	BA	True	False
15. B is preceded by A	BA	True	False
16. A follows B	AB	True	False
17. A does not follow B	BA	True	False
18. A is preceded by B	BA	True	False
19. B does not precede A	AB	True	False
20. B follows A	BA	True	False

Supplementary Figure S4 – Memory Recall

Memory Recall Task

When told to begin you will have 30 seconds to look over the patterns of numbers, objects, pictures, and shapes below. After 30 seconds you will be told to turn over the page.

When told to start writing you will be given 30 seconds to write down or draw as many of the objects as possible in the spaces provided (over the page). YOU ARE NOT TO REFER BACK TO THIS PAGE

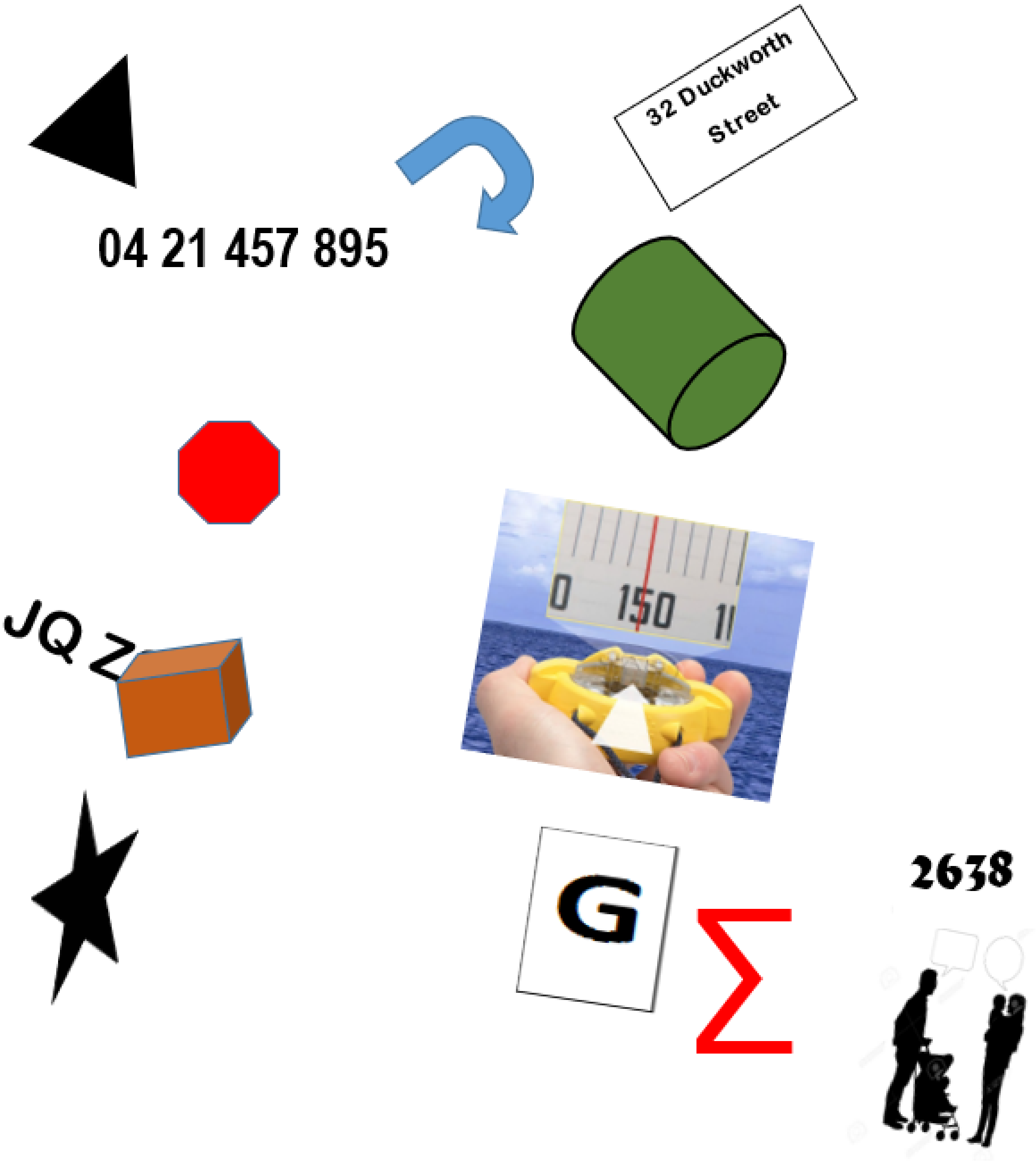

Supplement: Supplementary file 1 [file biology-11-00104-s001.zip › biology-1532988-supplementary.pdf]
